# Malaria smear positivity among Kenyan children peaks at intermediate temperatures as predicted by ecological models

**DOI:** 10.1186/s13071-019-3547-z

**Published:** 2019-06-06

**Authors:** Melisa M. Shah, Amy R. Krystosik, Bryson A. Ndenga, Francis M. Mutuku, Jamie M. Caldwell, Victoria Otuka, Philip K. Chebii, Priscillah W. Maina, Zainab Jembe, Charles Ronga, Donal Bisanzio, Assaf Anyamba, Richard Damoah, Kelsey Ripp, Prasanna Jagannathan, Erin A. Mordecai, A. Desiree LaBeaud

**Affiliations:** 1 Infectious Diseases and Geographic Medicine, Stanford University School of Medicine, Stanford, CA USA; 2 Department of Pediatrics, Division of Infectious Disease, Stanford University School of Medicine, Stanford, CA USA; 3 Centre for Global Health Research, Kenya Medical Research Institute, Kisumu, Kenya; 4 Department of Environment and Health Sciences, Technical University of Mombasa, Mombasa, Kenya; 5 Department of Biology, Stanford University, Stanford, CA USA; 6 Department of Pediatrics, Msambweni County Referral Hospital, Msambweni, Kenya; 7 Department of Pediatrics, Diani Health Center, Ukunda, Kenya; 8 RTI International, Washington, DC USA; 9 Epidemiology and Public Health Division, University of Nottingham, Nottingham, UK; 10 Universities Space Research Association (USRA), & NASA Goddard Space Flight, Biospheric Science Laboratory, Greenbelt, MD USA; 11 Morgan State University & NASA Goddard Space Flight, Biospheric Science Laboratory, Greenbelt, MD USA; 12 Department of Medicine, Hospital of the University of Pennsylvania, Philadelphia, USA; 13 Department of Pediatrics, Childrenʼs Hospital of Philadelphia, Childrenʼs Hospital of Philadelphia, USA

**Keywords:** Climate, Malaria, Kenya, Clinic-based surveillance

## Abstract

**Background:**

Ambient temperature is an important determinant of malaria transmission and suitability, affecting the life-cycle of the *Plasmodium* parasite and *Anopheles* vector. Early models predicted a thermal malaria transmission optimum of 31 °C, later revised to 25 °C using experimental data from mosquito and parasite biology. However, the link between ambient temperature and human malaria incidence remains poorly resolved.

**Methods:**

To evaluate the relationship between ambient temperature and malaria risk, 5833 febrile children (<18 years-old) with an acute, non-localizing febrile illness were enrolled from four heterogenous outpatient clinic sites in Kenya (Chulaimbo, Kisumu, Msambweni and Ukunda). Thick and thin blood smears were evaluated for the presence of malaria parasites. Daily temperature estimates were obtained from land logger data, and rainfall from National Oceanic and Atmospheric Administration (NOAA)’s Africa Rainfall Climatology (ARC) data. Thirty-day mean temperature and 30-day cumulative rainfall were estimated and each lagged by 30 days, relative to the febrile visit. A generalized linear mixed model was used to assess relationships between malaria smear positivity and predictors including temperature, rainfall, age, sex, mosquito exposure and socioeconomic status.

**Results:**

Malaria smear positivity varied between 42–83% across four clinic sites in western and coastal Kenya, with highest smear positivity in the rural, western site. The temperature ranges were cooler in the western sites and warmer in the coastal sites. In multivariate analysis controlling for socioeconomic status, age, sex, rainfall and bednet use, malaria smear positivity peaked near 25 °C at all four sites, as predicted *a priori* from an ecological model.

**Conclusions:**

This study provides direct field evidence of a unimodal relationship between ambient temperature and human malaria incidence with a peak in malaria transmission occurring at lower temperatures than previously recognized clinically. This nonlinear relationship with an intermediate optimal temperature implies that future climate warming could expand malaria incidence in cooler, highland regions while decreasing incidence in already warm regions with average temperatures above 25 °C. These findings support efforts to further understand the nonlinear association between ambient temperature and vector-borne diseases to better allocate resources and respond to disease threats in a future, warmer world.

**Electronic supplementary material:**

The online version of this article (10.1186/s13071-019-3547-z) contains supplementary material, which is available to authorized users.

## Background

The borders of the malaria belt in Africa are largely determined by climactic factors that limit suitability for both the *Anopheles* mosquito vector and the *Plasmodium* parasite [1]. Temperature, rainfall, and humidity affect the survival and transmission of malaria parasites. Aridity restricts *Anopheles* survival and ability for adult vectors to contribute to parasite transmission, explaining the lack of malaria north of the Saharan desert [2, 3]. Ambient temperature has also long been recognized as an important determinant of suitability limits for malaria transmission with effects on the life-cycle of the *Plasmodium* parasite and *Anopheles* mosquito vector [4–7]. The mosquito traits affecting malaria transmission include survival, abundance, feeding, development and competence. The parasite incubation rates and reproductive rates within the mosquito also affect malaria transmission [8]. As mosquitoes are cold-blooded ectotherms, temperature affects each of these traits and, in turn, malaria transmission intensity.

Early malaria models used linear estimates of mosquito and parasite physiology and estimated the thermal optimum for transmission at 31 °C [9, 10]; however, many of these biological processes are not linear, but instead are unimodal with a predicted optimum occurring at ambient temperatures regularly occurring in the environment. When using more accurate unimodal (hump-shaped) curves derived from temperature-controlled experiments to describe the relationship between temperature and mosquito survival, development, reproduction, biting, and egg laying rates, vector competence, and parasite development rate in the mosquito [4, 11–14], this transmission optimum was revised to 25 °C and malaria transmission was predicted to be bounded by 17 °C and 34 °C [15]. Previously, malaria risk was expected to broadly increase with warming climates; however, with peak transmission predicted at a lower ambient temperature, it is expected that climate warming will shift highly endemic areas to seasonal epidemics as suitability declines toward the upper thermal limit, and previously cooler, malaria free zones towards endemicity as temperatures approach the optimum [16]. These estimates suggest that small changes in temperature can dramatically alter the regions at risk for malaria transmission, and that impacts of climate warming on malaria transmission will not be uniform in magnitude or direction.

Despite the predicted relationship between ambient temperature and malaria transmission, clinical evidence linking the two has been limited. This is in part due to the difficulty of separating the role of temperature from other factors such as seasonal variation, malaria prevention interventions, treatment, and socioeconomic status. Prior studies have suggested that peak clinical malaria incidence occurs between 25 and 27 °C, in agreement with mechanistic modelling studies of malaria transmission [17]. Yet peak malaria transmission occurring at intermediate temperatures is not widely recognized, in part because data linking field malaria risk to ambient temperature are limited [15]. Malaria maps predicting changes in malaria intensity predict that the malaria belt will expand transmission further south, covering more of southern Africa, and will cause shifts from epidemic to endemic malaria transmission in regions such as in highland eastern Africa [16, 18]. Documenting a relationship between temperature and malaria incidence and the nonlinearity of this relationship is critical to predicting the impact of future climate warming on alterations in malaria incidence and endemicity.

In the present study, the effect of ambient temperature on malaria smear positivity was evaluated among children presenting with undifferentiated febrile illness to four heterogenous outpatient clinics in Kenya. In high endemic settings such as these, malaria smear positivity may be used as a proxy measure for incidence [19–22]. These clinical sites include a mix of urban and rural sites, local clinics and referral centers, and are climactically diverse with two sites in coastal Kenya and two sites in western Kenya.

## Results

### Characteristics of study sites and participants

**Fig. 1:**
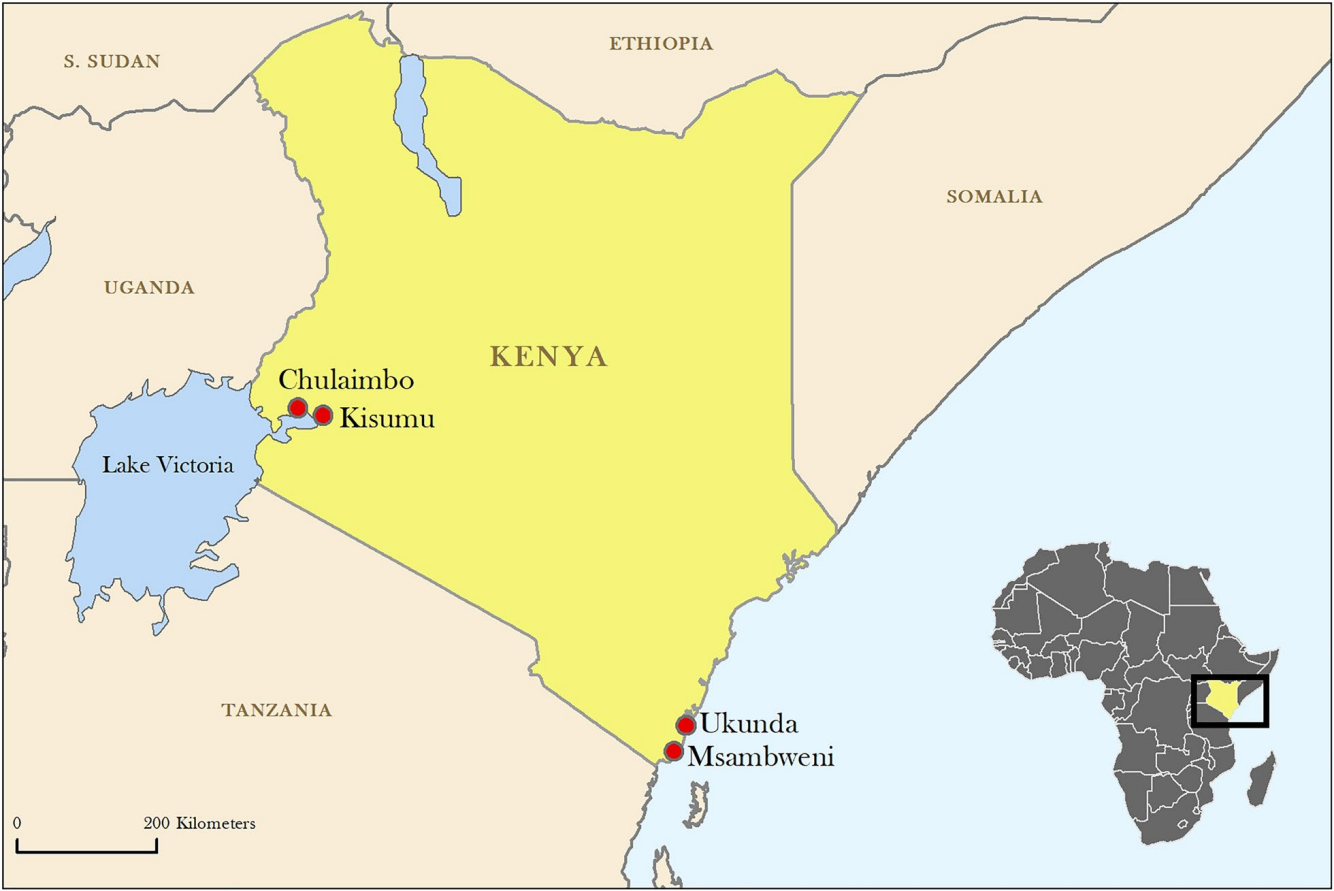
Map of study sites

**Fig. 2:**
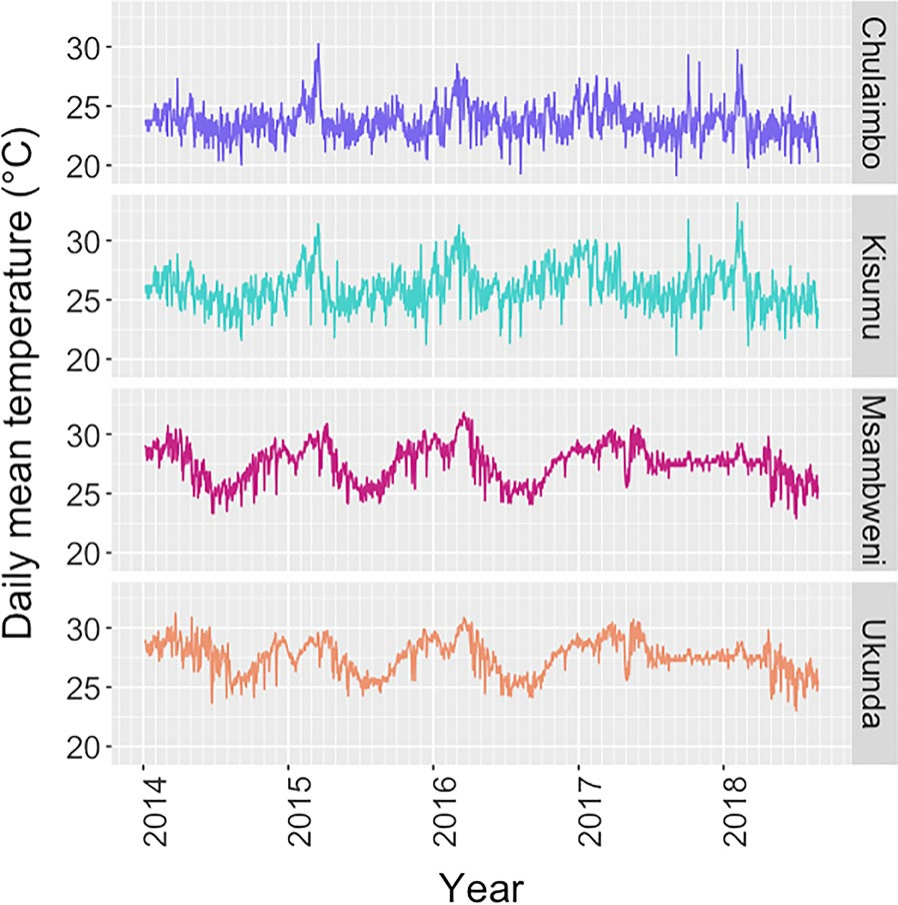
Temperature variation across four sites over the study period. The visit date is on the x-axis with minor ticks indicating months ranging from January 2014 to August 2018. Daily temperature means in °C are shown on the y-axis for each site: Chulaimbo (purple), Kisumu (teal), Msambweni (magenta), and Ukunda (orange)

Between June 2014 and August 2018, 5833 febrile children attending outpatient care were enrolled from four sites in western and coastal Kenya: 509 from Chulaimbo; 1177 from Kisumu; 2051 from Msambweni; and 2096 from Ukunda (Fig. 1). The western clinics in Chulaimbo and Kisumu are at higher altitude (1381 and 1131 meters above sea level, respectively) compared to the coastal sites, which are near sea level. The western sites had over two times higher 30-day cumulative rainfall compared to the coastal clinics (208 mm in Chulaimbo, 221 mm in Kisumu, 100 mm in Msambweni and 98 mm in Ukunda). The mean temperature and range in the 30 days prior to presentation was 23.8 °C (22.5–26.5 °C) in Chulaimbo, 26.1 °C (24.1–29.3 °C) in Kisumu, 27.5 °C (24.9–31.0 °C) in Msambweni, and 27.5 °C (25.2–30.2 °C) in Ukunda. Seasonal variation in temperature occurred at all sites (Fig. 2). The percentage of children always using bednets was lowest in Kisumu (41.9%). Over 97% of children from Chulaimbo reported mosquito bites within the last four weeks. Children visiting the clinic in Kisumu had the highest rates of electricity in the home, flush toilets, cement floors, and piped water. The distribution of assets and other home characteristics varied across sites (Table 1).

**Table 1:** Characteristics of enrolled patients, location and climate, and socioeconomic indicators at four outpatient Kenyan clinic sites

	Chulaimbo^a^	Kisumu^b^	Msambweni^c^	Ukunda^d^
Enrolled patients				
No. enrolled (January 2014–August 2018)	509	1177	2051	2096
Malaria smear positivity (%)	83.1	42.2	41.7	49.3
Female sex (%)	47.2	47.4	47.0	47.6
Report mosquito bites during last 4 weeks (%)	97.2	81.2	66.4	91.9
Always uses bednet (%)	67.2	41.9	89.2	82.7
Mean age (range) (years)	6.1 (0–17)	3.9 (0–15)	5.0 (0–17)	8.2 (0–17)
Location and climate				
Location	Western Kenya	Western Kenya	Coastal Kenya	Coastal Kenya
Rural/urban	Rural	Urban	Rural	Urban
Altitude (m)	1381	1131	23	14
Cumulative mean 30-day rainfall (mm)	208	221	100	98
Mean 30-day temperature °C (range)	23.8 (22.5–26.5)	26.1 (24.1–29.3)	27.5 (24.9–31.0)	27.5 (25.2–30.2)
Socioeconomic indicators				
Iron roof in home (%)	96.4	98.0	31.8	54.2
Predominant water source (%)	46.9 River/pond	87.1 Tap/piped water	72.9 Well/borehole	76.6 Well/borehole
Predominant latrine type (%)	97.0 VIPL	83.9 VIPL	54.0 PL	83.3 VIPL
Earthen floor (%)	68.3	18.5	62.7	47.1
Electricity in home (%)	15.2	75.8	31.2	31.4
Domestic worker in home (%)	2.8	4.3	3.9	1.4
Family owns bicycle (%)	15.0	20.3	34.0	35.2
Family owns telephone (%)	89.6	97.4	82.0	75.8
Family owns radio (%)	66.1	73.3	41.6	58.3
Family owns motor vehicle (%)	6.6	15.9	12.0	6.7
Family owns television (%)	19.4	64.4	18.9	32.1

^a^Chulaimbo County Hospital and Mbaka Oromo Dispensary

^b^Obama Children’s Hospital in Jaramogi Oginga Odinga Teaching and Referral Hospital

^c^Msambweni County Hospital

^d^Diani Health Centre

*Abbreviations*: PL, Pit latrine; VIPL, ventilated improved pit latrine

### Malaria smear positivity across diverse clinical sites in Kenya

Malaria smear positivity was highest in Chulaimbo (83.1%) compared with the other three sites (range: 41.7–49.3%) (Table 1). Overall, febrile children with malaria parasitemia were older than smear-negative children (6.3 years *vs* 5.7 years; t-test: *t*_(5724)_ = 5.7, *P* < 0.001). Children with malaria had significantly lower socioeconomic indicators including roof type, floor type, water source and latrines. Smear negative children were significantly more likely to have electricity at home (44 *vs* 21%, Chi-square test: *χ*^2^ = 300.45, *df* = 1, *P* < 0.001). At all four sites combined, reported bednet use (Chi-square test: *χ*^2^ = 5.98, *df* = 1, *P* = 0.01) and mosquito bites within the past four weeks (Chi-square test: *χ*^2^ = 51.26, *df* = 1, *P* < 0.001) were associated with smear positivity. Smear positive children were more likely to be female than smear negative children (48.8 *vs* 46.0%, Chi-square test: *χ*^2^ = 4.59, *df* = 1, *P* = 0.03). In multivariate analysis of all four sites, age above four years was associated with increased risk of smear positivity compared to the zero to four age range (Table 2). Low socioeconomic status as defined by a six-point wealth index and not always using a bednet was also associated with smear positivity in multivariate analysis (Table 2). Sex and rainfall were not significant predictors of malaria smear positivity in the final model (Table 2).

**Table 2:** Predictors of malaria smear positivity using a multivariate model

	OR (95% CI)	*P*-value
Lagged 30-day mean temperature^a^		
<24 °C	1.05 (0.56–2.00)	0.87
24–26 °C	1.97 (0.43–9.02)	0.38
>26 °C	0.31 (0.18–0.52)	<0.001
Lagged 30-day cumulative rainfall	1.01 (0.96–1.07)	0.59
Age categories (years)		
≤4	Ref	
>4 and ≤8	1.39 (1.22–1.60)	<0.001
>8 and ≤12	1.59 (1.33–1.90)	<0.001
>12	1.34 (1.09–1.66)	<0.001
Always uses bednet	0.83 (0.72–0.96)	0.01
Low wealth index^b^	1.50 (1.34–1.69)	<0.001
Female sex	1.11 (1.0–1.24)	0.05

^a^Temperature was included as a nonlinear predictor, and the effect of temperature alone on the odds of malaria smear positivity is included in Additional file 1: Figure S2

^b^Defined as household having less than 3 of the following: domestic worker, bicycle, telephone, radio, motor vehicle and bicycle

### Associations between temperature and malaria smear positivity, stratified by site

**Fig. 3:**
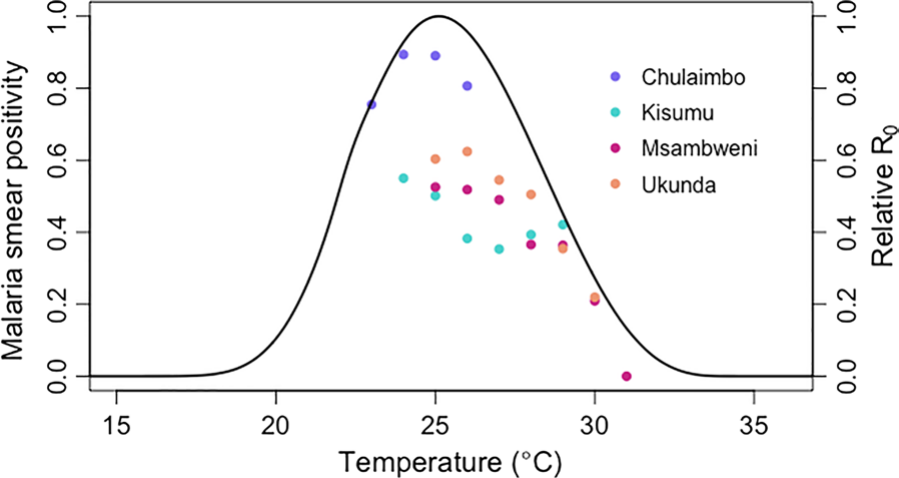
Malaria smear positivity by ambient temperature with relative reproductive number (R_0_) curve. Points represent the average smear positivity rate over temperature (1 °C intervals of temperature on the x-axis) for Chulaimbo (purple), Kisumu (teal), Msambweni (magenta), and Ukunda (orange). Line represents predicted basic reproductive number (R_0_, rescaled to range from zero to one) as a function of temperature from an independent, *a priori* ecological model derived from laboratory experimental data [15]

**Fig. 4:**
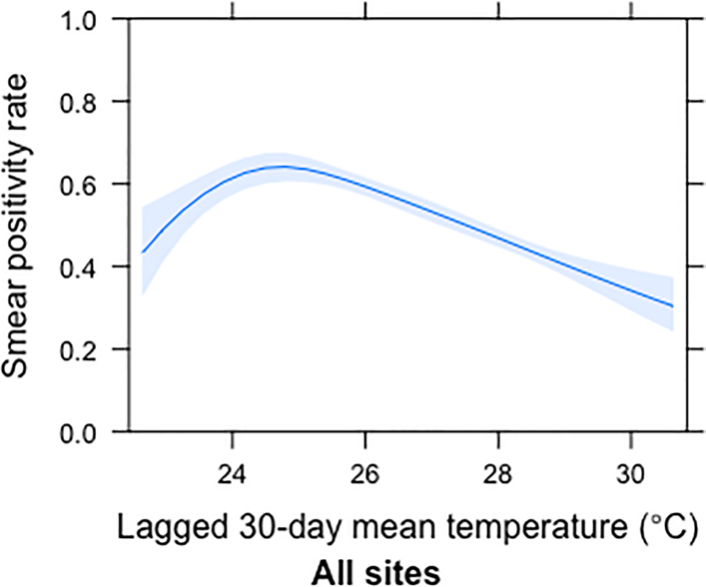
Effect of 30-day lagged mean temperature on malaria smear positivity at all four sites combined after controlling for clinic site, sex, rainfall, year, age category, bednet use and socioeconomic status. The x-axis displays the mean 30-day temperature in °C lagged by 30 days relative to the date of visit and the y-axis is the probability of malaria smear positivity

Malaria smear positivity peaked near 25 °C at all four sites with decreasing smear positivity past 25 °C when grouped by 1 °C (Fig. 3). Additionally, smear positivity followed the unimodal trajectory of the reproductive number curve which was predicted *a priori* by an ecological model [15]. In Chulaimbo, smear positivity increased at temperatures below 25 °C but then declined (Fig. 3). In multivariate analysis, controlling for clinic site, sex, rainfall, year, age category, bednet use, and socioeconomic status, lagged 30-day mean temperature above 26 °C was significantly associated with a decreased risk of smear positivity compared to lower temperatures (OR: 0.31, 95% CI: 0.18–0.52, *P* < 0.001). Adjusted malaria smear positivity controlling for these covariates is unimodal with a peak near 25 °C (Fig. 4).

## Discussion

Temperature has long been recognized as an important determinant of malaria risk via its effects on mosquitoes, yet the precise role of temperature in human malaria incidence has been debated. We present evidence that ambient temperature was associated with malaria smear positivity in four heterogeneous outpatient clinics in western and coastal Kenya with endemic malaria. Malaria smear positivity in this study peaked near 25 °C, and estimates remained even after adjustment for the effects of site, sex, rainfall, year, age, bednet use and socioeconomic status. Together, these data are consistent with those predicted by ecological models derived from independent data from laboratory experiments [15].

Historically, malaria risk was expected to broadly increase with global warming because prior models placed the optimal temperature for malaria transmission around 31 °C [9, 10, 23]. Conflicting results on the effect of climate change and temperature on malaria transmission in previous work emerged due to the concomitant effects of rainfall, treatment availability, and preventative interventions on malaria transmission. Additionally, when control measures interrupt malaria transmission in areas where climate conditions are suitable, predicting suitability limits for transmission becomes difficult. More recently, ecological modelling studies suggested that the prior estimates for optimal temperatures for malaria transmission were too high because they did not incorporate nonlinear effects of temperature on multiple mosquito and parasite traits [15]. Similarly, observational studies showed increasing malaria transmission in highland regions of East Africa but declining rates in endemic regions and overall brought into question the predicted impact of climate change on malaria incidence [5, 24].

With increasing evidence that climate change will impact malaria transmission in a nuanced way, new maps predicting future malaria risk have been proposed [16]. Despite significant advances in ecological models, there have been few studies that directly assess the effect of ambient temperature on human malaria incidence. The data presented here allow us to assess the risk of malaria smear positivity based on land temperature data and satellite rainfall data at four heterogeneous clinic sites in Kenya. All of these sites are considered highly endemic regions for malaria, hence making the use of malaria smear positivity an appropriate proxy for malaria incidence [19–21].

Overall, this study demonstrates high smear positivity rates in both coastal and western Kenya. The temperature differential in coastal *versus* western Kenya allowed us to evaluate temperature effects in two areas with markedly different climates and altitude. Sites near Lake Victoria in western Kenya experience cooler temperatures and are at higher altitude than sites in coastal Kenya. Nevertheless, the combined data indicate that malaria smear positivity peaks at temperatures near 25 °C, corresponding to the thermal optima for malaria transmission predicted by ecological models (Fig. 4). Chulaimbo was the only site with temperatures below 24 °C which allowed for visualization of the unimodal relationship of temperature on malaria incidence. At all four sites, the effect of ambient temperature on malaria risk supported predictions from the ecological models, even after controlling for rainfall. Based on these data, we infer that increasing temperatures by 1 °C, for example, would result in unchanged levels of malaria in the west but decreased malaria incidence on the coast of Kenya.

These results support previous predictions from ecological models that optimal malaria transmission occurs around 25 °C, which implies that climate warming should bring cooler regions closer to the optimal temperature for transmission and, conversely, may decrease transmission in areas with increasing temperatures above 25 °C [15, 16]. As climate change pushes temperatures closer to 25 °C, vector control and malaria prevention may prove more difficult in some currently cooler areas, whereas areas that are currently highly endemic with mean temperatures around 25 °C may experience decreased temperature suitability and shifts in seasonality for malaria. The 2014 Intergovernmental Panel on Climate Change predicted that global surface temperature change will exceed 1.5 °C by the end of the 21st century relative to 1850–1900 [25]. These predictions have important implications for malaria control and transmission given the rapid decrease in malaria smear positivity after temperatures increase above 27 °C in this study. Many model-based malaria maps have predicted future malaria spread with climate warming: it is critical to update these predictions based on our field observation that malaria smear positivity decreases with increasing temperature above 25 °C, in concordance with ecological models. These malaria transmission projections are crucial for planning control strategies and allocation of resources with ongoing climate change.

Ambient temperature conditions affect both mosquito life-cycle and the *Plasmodium* extrinsic incubation period. The thermal optima and limits for different transmission-relevant traits vary by mosquito and parasite species. In particular, for *Aedes aegypti*, the vector of dengue and chikungunya viruses, among others, transmission potential peaks at 29 °C, implying that climate warming could expand suitability for arboviruses in the same sites where malaria transmission declines [26]. In fact, we found non-malaria undifferentiated febrile illness increased above 27 °C, which could be explained by declining malaria and increasing arboviral infections such as dengue virus and chikungunya virus. In our cohort, dengue and chikungunya are hyperendemic [27] and as malaria rates decline in coastal Kenya with rising temperature, it is possible that dengue and chikungunya risk may increase.

There were several limitations to this study. The use of various measures of temperature can influence the findings on effects of temperature on malaria transmission. We used 30-day mean temperature and cumulative rainfall lagged by 30 days as individual transmission-relevant and time-integrated metrics for this study. However, temperature varies on daily, weekly, seasonal, and interannual scales, and other studies account for this variation in different ways. Paaijmans et al. [28] suggest that daily temperature variation is important for malaria transmission, a potentially important avenue for future clinical studies, but we expect such variability is more important at temperature extremes than at the optimum, which was the focus of this study. The source of ambient temperature measurements may also impact findings on the effect of temperature on malaria transmission, as remotely sensed land surface temperatures that are widely available measure radiative skin temperature of the land surface which differs from ambient temperatures collected *in situ*. In our study, we found that a lagged 30-day average daily temperature was correlated with smear positivity, when we appropriately accounted for the nonlinear relationship between temperature and malaria incidence. Thus, it could be important to appropriately adjust remotely sensed land surface temperature measurements in future research. Further research is needed to understand other measures of ambient temperature on malaria risk, and clinical data from sites with a greater range of temperatures below the thermal optimum would help verify our findings across a greater range of temperatures. Finally, the potential for malaria vectors and parasites to adapt to warming temperatures remains a critical empirical gap [29].

While there have been significant reductions in malaria transmission over the past decade, there is also evidence of changing trends in malaria endemicity geographically [30]. Changing risk for malaria is partly explained by interventions such as long-lasting insecticidal nets, indoor residual spraying, and artemisinin-based treatment, but may also be affected by changes in climatic factors [31]. However, the potential for increasing temperatures to reduce malaria incidence in warm, endemic locations has not been rigorously investigated, in part because the nonlinear effects of temperature on transmission have not been widely recognized. To our knowledge, this study provides some of the first field evidence of a unimodal relationship between temperature and human malaria incidence, with a peak at 25 °C, as predicted by ecological models fit from laboratory experimental data. More widespread consideration of the fundamentally nonlinear relationship between temperature and vector transmission, not just for malaria but for all vector-borne diseases, is critical for anticipating and responding to changes in disease burden under changing climates. Applying more accurate thermal physiology is a fundamental building block for incorporating and predicting the simultaneous influence of changes in population density, migration, urbanization, socioeconomic conditions, trade and travel, species invasions, and other rapid ongoing global changes that impact vector-borne disease. Understanding the association between ambient temperature and malaria transmission will become increasingly important in a future, warmer climate to predict necessary changes in the allocation of prevention and treatment efforts.

## Conclusions

This study provides direct field evidence of a unimodal relationship between ambient temperature and human malaria incidence with a peak malaria transmission occurring at lower temperatures than previously recognized, as predicted by *a priori* ecological models. These findings support efforts to further understand the nonlinear association between ambient temperature and vector-borne diseases to better allocate public health resources and to respond to disease threats in a future, warmer world.

## Methods

### Study sites and participants

This study consisted of a cohort of children less than 18 years of age from four study areas with heterogenous malaria transmission in Kenya: Chulaimbo, Kisumu, Msambweni and Ukunda (Fig. 1). The official names of the five health facilities are: Chulaimbo County Hospital and Mbaka Oromo Dispensary (both in Chulaimbo); Jaramogi Oginga Odinga Teaching and Referral Hospital (where Obama Children’s Hospital is a wing) (Kisumu); Msambweni County Hospital (Msambweni) and Diani Health Centre (Ukunda). The sites have been described previously [32, 33]. Briefly, Chulaimbo and Kisumu are located in the Lake Victoria region. The Kisumu site is a referral hospital in an urban setting, whereas the Chulaimbo site is in a rural setting. Msambweni District Hospital and Ukunda/Diani Health Center are in coastal Kenya. Msambweni is a district hospital in a rural setting and Diani Health Centre is a clinic in an urban setting. These sites were chosen to provide geographical diversity (west and coast) and both rural and urban locations. The current study used data collected from an acutely ill, febrile cohort attending outpatient care from January 6, 2014 to August 27, 2018.

### Study procedures and follow-up

Children who presented with an acute febrile illness (temperature greater than 38 °C or reported fever) and no localizing signs or symptoms were enrolled at one of the four study sites. Children with localizing illness (i.e. traumatic injury, acute pneumonia and urinary tract infections) were not included. Participants were consented and underwent a detailed clinical history and physical examination by a certified clinical officer. In addition, indicators of socioeconomic status and mosquito exposure were collected. Blood was collected by phlebotomy from each of the study participants. A thick and thin blood smear for malaria parasite examination was prepared for all participants, stained with 2% Giemsa and read by a central laboratory technologist in each region (coast and west). Children were treated with artemether-lumefantrine based on standardized Kenyan Ministry of Health protocols and referred for hospitalization for severe illness. Data were collected using open data kit (ODK) and stored in REDCap [34].

### Climate data

Two temperature loggers (HOBO^®^ Onset data loggers, Onset Computer Corporation 470 Bourne, MA, USA) were installed under the eaves of two houses within each of the four study areas: Chulaimbo, Kisumu, Msambweni and Ukunda. Data was recorded hourly. Daily temperature means were obtained from the land logger data and missing data were imputed from logger data obtained from the paired site where possible and otherwise imputed with publicly available data from Weather Underground (www.wunderground.com; weather station codes for the coastal and western sites are HKMO and HKKI respectively). Missing data were imputed by adjusting available data from the paired site or Weather Underground by the slope and intercept of a linear regression equation based on the relationship between the two datasets (blue lines in Additional file 1: Figure S1). This study included children enrolled between January 6, 2014 and August 27, 2018, a span of 1695 days. There were 141 missing days from the HOBO loggers in Kisumu and Chulaimbo and these were filled in by Weather Underground data. For Msambweni, there were 872 days of missing records from the HOBO logger, 628 of these measurements were filled with land logger data from Ukunda given the close geographical proximity. An additional 244 daily temperature readings from Msambweni were filled in by Weather Underground data. In Ukunda, there were 297 missing daily temperature data from the HOBO land loggers with 53 filled by Msambweni HOBO records and 244 filled by Weather Underground data. The correlation between HOBO records and Weather Underground data was visualized for all sites (see Additional file 1: Figure S1). For rainfall, all measurements were taken from National Oceanic and Atmospheric Administration (NOAA)’s Africa Rainfall Climatology (ARC) data at 0.1° × 0.1° spatial resolution [35]. The ARC dataset is produced using a combination of rainfall gauge measurements and METEOSAT satellite data to provide gridded rainfall estimates. Thirty-day mean temperature and 30-day cumulative rainfall were estimated and each lagged by 30 days, relative to the febrile visit.

### Statistical analysis

Univariate analysis using chi-square tests for categorical variables and t-tests for continuous variables was used to evaluate the association between malaria smear positivity and indicators of mosquito exposure, socioeconomic status, sex, rainfall, temperature, and age at all four sites combined. Factors that were significant and relevant in univariate analysis were included in multivariate analysis. Relationships between the primary outcome, malaria smear positivity, and temperature were modeled using R statistical language package *lme4* [36] and *splines* [37] with a generalized linear mixed model fit by maximum likelihood. Model diagnostics were run using R statistical language package *DHARMa* [38] and showed uniform distributions of residuals. We allowed for a nonlinear relationship between the outcome and 30-day lagged temperature using natural cubic splines with knots placed at 24 °C and 26 °C. Covariates included in multivariate analysis included socioeconomic status, sex, age in four-year categories, lagged 30-day cumulative rainfall and bednet use. Multicollinearity was tested using the variance inflation factor (VIF) (Additional file 1: Table S1) and explanatory power of the final model was assessed using a pseudo R2 estimate (Additional file 1: Table S2). The age categories used were: <4 years; >4 and ≤8 years; >8 and ≤12 years; and >12 years. Year of visit and site were included as random intercepts. We characterized socioeconomic status with a six-point wealth index based on the child’s family having a domestic worker, bicycle, telephone, radio, motor vehicle, or television. Households with less than three of the components were defined as having a low wealth index. Data were analyzed and visualized using RStudio statistical software version 1.0.143.

## Additional file

**Additional file 1: Figure S1.** Correlation of HOBO logger temperature data between nearest clinical sites and with Weather Underground data. Left panel: comparison of HOBO logger temperature data between nearest clinical site (top: Msambweni and Ukunda; bottom: Kisumu and Chulaimbo). Middle and right panels: comparison of HOBO logger temperature data at a clinical site and Weather Underground data from the nearest weather station (weather station code for Msambweni and Ukunda is HKMO and for Kisumu and Chulaimbo is HKKI). Dashed black lines indicate the regression line where y = x; blue lines indicate the linear regression between the two data sets (y = mx + b). The linear regression equations (blue lines) were used to adjust source data to fill in missing data. **Figure S2.** The nonlinear effect of temperature on malaria smear positivity. The plot shows the nonlinear effect of temperature alone on the odds of malaria smear positivity using a structured additive regression model (*R2BayesX* R package). The x-axis shows temperature and the y-axis shows the odds ratio of malaria smear positivity. The red lines indicate the 95% confidence intervals. The areas above the green line indicate odds ratios above one. **Table S1.** Evaluation of multicollinearity. Variance inflation factors (VIF) for our final model show no evidence of multicollinearity between predictors, with VIF > 4 as evidence of multicollinearity (R package *MuNIn*). **Table S2.** Explanatory power of the generalized linear mixed model. The first column is marginal pseudo-*R*^2^ which describes the explanatory power of the fixed effects. The second column is the conditional pseudo-*R*^2^ which describes the full model (random and fixed effects). The rows indicate different methods of estimation. The full model explains 15–18% of variation in the outcome (R package *car*).

## Data Availability

Data supporting the conclusions of this article are provided within the article and its additional file. The datasets used and analyzed during the present study are available from the corresponding author on reasonable request.
