# Supplementary material for: Malaria smear positivity among Kenyan children peaks at intermediate temperatures as predicted by ecological models
Source: Parasit Vectors. 2019 Jun 6;12:288. doi: 10.1186/s13071-019-3547-z (PMC6555721; doi:10.1186/s13071-019-3547-z)
Supplement: Supplementary file 1 — Additional file 1: Figure S1. Correlation of HOBO logger temperature data between nearest clinical sites and with Weather Underground data. Left panel: comparison of HOBO logger temperature data between nearest clinical site (top: Msambweni and Ukunda; bottom: Kisumu and Chulaimbo). Middle and right panels: comparison of HOBO logger temperature data at a clinical site and Weather Underground data from the nearest weather station (weather station code for Msambweni and Ukunda is HKMO and for Kisumu and Chulaimbo is HKKI). Dashed black lines indicate the regression line where y = x; blue lines indicate the linear regression between the two data sets (y = mx + b). The linear regression equations (blue lines) were used to adjust source data to fill in missing data. Figure S2. The nonlinear effect of temperature on malaria smear positivity. The plot shows the nonlinear effect of temperature alone on the odds of malaria smear positivity using a structured additive regression model (R2BayesX R package). The x-axis shows temperature and the y-axis shows the odds ratio of malaria smear positivity. The red lines indicate the 95% confidence intervals. The areas above the green line indicate odds ratios above one. Table S1. Evaluation of multicollinearity. Variance inflation factors (VIF) for our final model show no evidence of multicollinearity between predictors, with VIF > 4 as evidence of multicollinearity (R package MuNIn). Table S2. Explanatory power of the generalized linear mixed model. The first column is marginal pseudo-R2 which describes the explanatory power of the fixed effects. The second column is the conditional pseudo-R2 which describes the full model (random and fixed effects). The rows indicate different methods of estimation. The full model explains 15–18% of variation in the outcome (R package car). [file 13071_2019_3547_MOESM1_ESM.docx]

**Additional file 1**


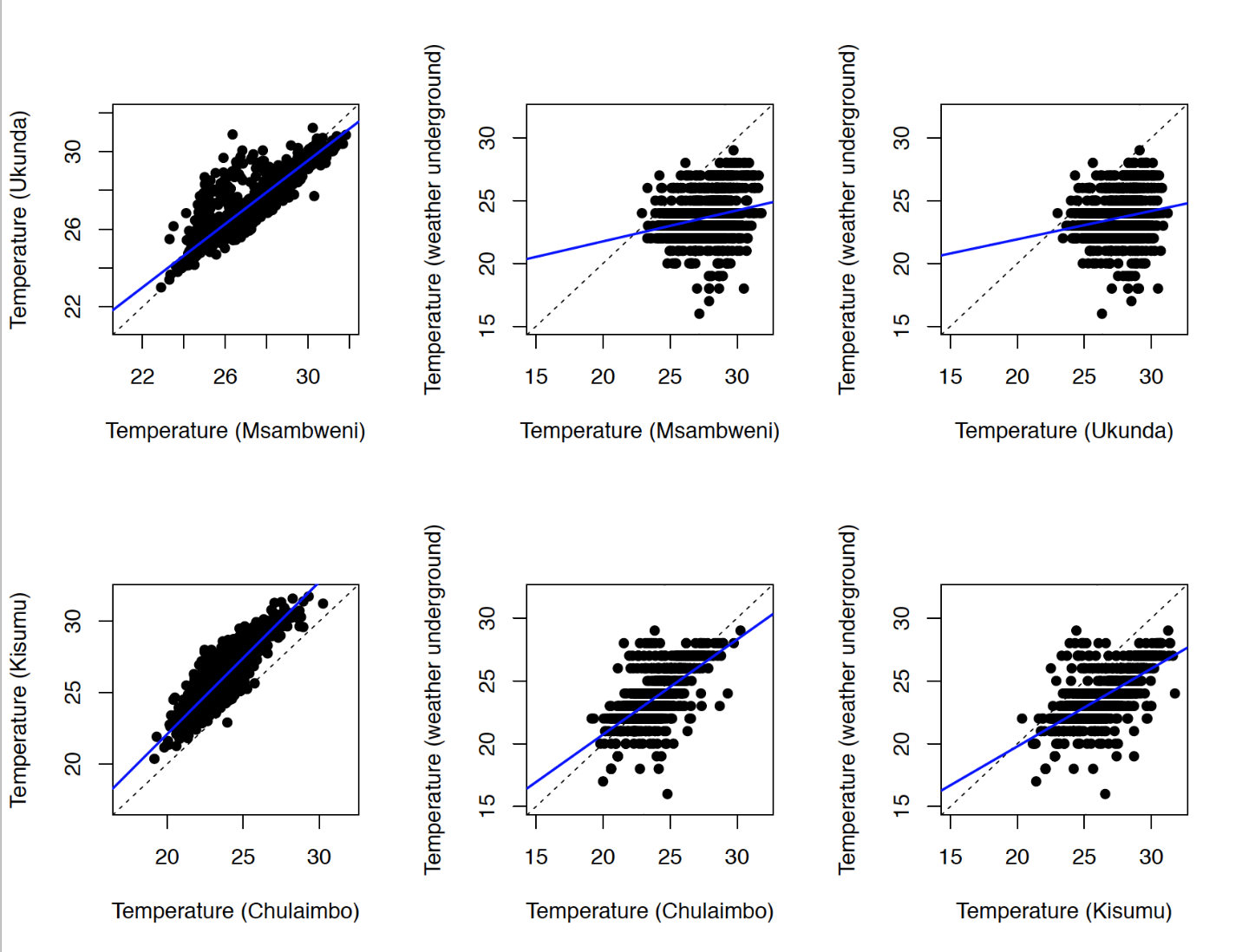


**Additional file 1: Figure S1.** **Correlation of HOBO logger temperature data between nearest clinical sites and with Weather Underground data.** Left panel: comparison of HOBO logger temperature data between nearest clinical site (top: Msambweni and Ukunda; bottom: Kisumu and Chulaimbo). Middle and right panels: comparison of HOBO logger temperature data at a clinical site and Weather Underground data from the nearest weather station (weather station code for Msambweni and Ukunda is HKMO and for Kisumu and Chulaimbo is HKKI). Dashed black lines indicate the regression line where y=x; blue lines indicate the linear regression between the two data sets (y=mx+b). The linear regression equations (blue lines) were used to adjust source data to fill in missing data.


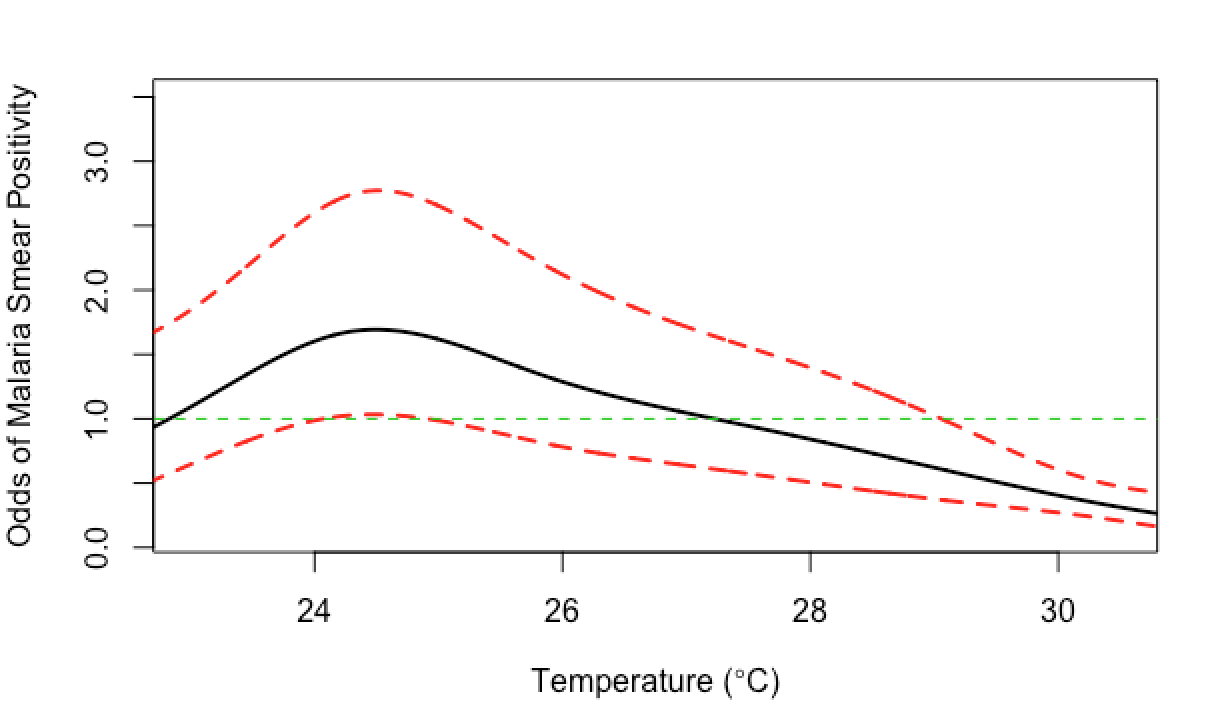


**Additional file 1:** **Figure S2. The nonlinear effect of temperature on malaria smear positivity.**

This plot shows the nonlinear effect of temperature alone on the odds of malaria smear positivity using a structured additive regression model (R2BayesX R package). The x axis shows temperature and the y axis shows the odds ratio of malaria smear positivity. The red lines indicate the 95% confidence intervals. The areas above the green line indicate odds ratios above one.

**Additional file 1: Table S1. Evaluation of Multicollinearity.** Variance inflation factors (VIF) for our final model shows no evidence of multicollinearity between predictors, with VIF > 4 as evidence of multicollinearity (R package “MuNIn”).

|  | GVIF | Df |
| --- | --- | --- |
| Lagged 30-day Temperature | 1.09 | 3 |
| Lagged 30-day Cumulative Rainfall | 1.08 | 1 |
| Age | 1.02 | 3 |
| Bednet Use | 1.02 | 1 |
| Socioeconomic Status | 1.02 | 1 |
| Sex | 1.00 | 1 |

**Additional file 1:** **Table S2. Explanatory power of the generalized linear mixed model.** The first column is marginal pseudo R^2^ which describes the explanatory power of the fixed effects. The second column is the conditional pseudo R^2^ which describes the full model (random and fixed effects). The rows indicate different methods of estimation. The full model explains 15-18 % of variation in the outcome (R package “car”).

|  | Marginal R^2^ | Conditional R^2^ |
| --- | --- | --- |
| Theoretical | 0.05 | 0.19 |
| Delta | 0.04 | 0.16 |
